# Partial Absence of Pleuropericardial Membranes in *Tbx18*- and *Wt1*-Deficient Mice

**DOI:** 10.1371/journal.pone.0045100

**Published:** 2012-09-11

**Authors:** Julia Norden, Thomas Grieskamp, Vincent M. Christoffels, Antoon F. M. Moorman, Andreas Kispert

**Affiliations:** 1 Institut für Molekularbiologie, OE5250, Medizinische Hochschule Hannover, Hannover, Germany; 2 Department of Anatomy, Embryology and Physiology, Academic Medical Center, University of Amsterdam, Amsterdam, The Netherlands; Integrated Research Centre, Germany

## Abstract

The pleuropericardial membranes are fibro-serous walls that separate the pericardial and pleural cavities and anchor the heart inside the mediastinum. Partial or complete absence of pleuropericardial membranes is a rare human disease, the etiology of which is poorly understood. As an attempt to better understand these defects, we wished to analyze the cellular and molecular mechanisms directing the separation of pericardial and pleural cavities by pleuropericardial membranes in the mouse. We found by histological analyses that both in *Tbx18*- and *Wt1*-deficient mice the pleural and pericardial cavities communicate due to a partial absence of the pleuropericardial membranes in the hilus region. We trace these defects to a persisting embryonic connection between these cavities, the pericardioperitoneal canals. Furthermore, we identify mesenchymal ridges in the sinus venosus region that tether the growing pleuropericardial membranes to the hilus of the lung, and thus, close the pericardioperitoneal canals. In *Tbx18-*deficient embryos these mesenchymal ridges are not established, whereas in *Wt1-*deficient embryos the final fusion process between these tissues and the body wall does not occur. We suggest that this fusion is an active rather than a passive process, and discuss the interrelation between closure of the pericardioperitoneal canals, lateral release of the pleuropericardial membranes from the lateral body wall, and sinus horn development.

## Introduction

The lung and the heart, the two major organs of the chest, reside in separate compartments of the thoracic cavity. The lobes of the lung are located in two pleural cavities that occupy the lateral aspect of the thoracic cavity; the heart is located within the pericardial cavity, which, in turn, occupies the medial region of the mediastinum, an entity of loose connective tissue that harbors all other chest organs in the midline. Pleural and pericardial cavities are tiny fluid filled spaces that are lined by thin mesothelial linings, the pleura and pericardium, folding back on themselves at the hilus of the organ. Their inner or visceral layer covers the organ and its associated structures, thus allowing protection and mobility. The outer or parietal layer provides anchorage and sealing off from adjacent compartments. The parietal layer of the pleura is largely attached to the body wall, whereas the parietal pericardium is only locally attached to the sternum and diaphragm, but largely aligns with the free aspect of the parietal pleura to form a double-layered wall that is further stiffened by deposition of fibrous material between the two epithelia. These pleuropericardial membranes (PPMs) anchor the heart in the mediastinum and separate the pericardial from the pleural cavities [1].

Congenital absence of the PPMs (often simply referred to as “pericardial absence” in clinical terminology) is a rare condition of unknown molecular basis. In the majority of the cases the PPM of the left side is completely missing. Patients with such a condition are either asymptomatic or have non-specific chest pain, and are without need for treatment. In contrast, patients with partial absence of the PPMs are at risk for herniation and fatal strangulation of the heart [2].

(Partial) absence of PPMs is generally interpreted as a persisting embryonic situation in which the pleural and pericardial cavities were not yet separated [2], [3]. In fact, the post-gastrulation stage embryo initially harbors only a single large intraembryonic cavity that is lined by the visceral and parietal layers of the lateral plate mesoderm, the splanchnopleura and the somatopleura, respectively. This cavity is gradually partitioned into the peritoneal cavity and the thoracic cavity by the ingrowth of a tissue bridge at the level of the liver, the septum transversum, from which the diaphragm will derive. More anteriorly, the PPMs split off the lateral body wall concomitant with the expansion of the lung and the future pleural cavity. Irrespective of the fact whether these PPMs arise as short folds that protrude into the thoracic cavity as suggested by some authors, PPMs harbor the cardinal veins (Ductus cuvieri) and are further stretched upon cardiac looping and the associated relocalization of the cardinal veins towards the midline. PPMs finally reach the hilus of the lung to obliterate the remaining communication area between the cavities, the bilateral pericardioperitoneal canals (PPCs). Histological analyses of human embryos suggested that fusion of the PPMs, thus the complete separation of the pleural and pericardial cavities, is a mere passive consequence of the complex morphogenetic movements of cardinal veins and the heart within the growing thoracic cavity, whereas other authors argue for an active fusion process between the PPMs and the lining of the lung hilus [4].

**Figure 1:**
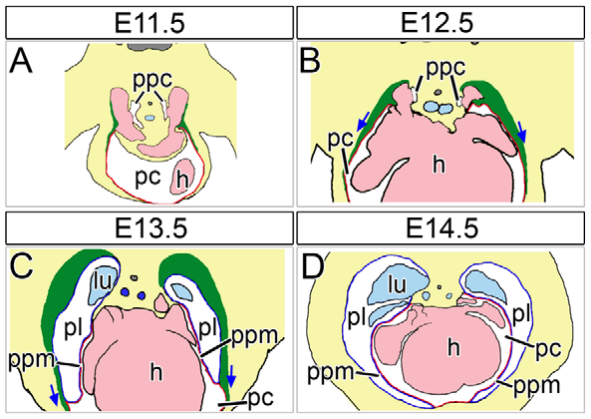
Schematic diagram of murine PPM development. (A–D) Scheme of PPM development based on transverse sections through the cardiac venous pole of wildtype embryos from E11.5 to E14.5. The lumen of the caval veins and the heart is marked in pale pink, the lung and trachea in pale blue, the (parietal) pleura in blue, the (parietal) pericardium in red, and the degenerating subcoelomic mesenchyme in green. Blue arrows demonstrate the direction of the detachment of the PPMs from the subcoelomic mesenchyme. Note the PPMs are double-layered consisting of the parietal layer of the pleura and the pericardium. Dorsal is up, ventral is down. h, heart; lu, lung; pc, pericardial cavity; pl, pleural cavity; ppc, pericardioperitoneal canal; ppm, pleuropericardial membranes.

To resolve the cellular processes and define the molecular circuits that drive the formation of PPMs and of separated pleural and pericardial cavities, the characterization of PPM development in wildtype mice and the analysis of mice that exhibit partial pericardial absence would undoubtedly be of great value. We have recently shown that PPMs are gradually released from the subcoelomic mesenchyme between embryonic day (E) 11.5 and E14.5, and that distinct pleural and pericardial cavities are established at around E13.5 in the mouse (Figure 1). Moreover, the transcription factor encoded by the Wilms tumor 1 gene (*Wt1*) and downstream retinoic acid signaling were characterized as first crucial players of PPM release by regulation of local apoptosis in the subcoelomic mesenchyme, and of myocardialization and medial re-localization of cardinal veins [5].

Furthermore, we showed that *T-box (Tbx)18*, a member of the conserved family of T-box transcription factors, is expressed in a specialized precursor cell population, which is needed for the formation of the sinus node and myocardialization and positioning of the cardinal veins within the PPMs [6], [7].

Here, we show that *Tbx18-* and *Wt1*-deficient mice additionally exhibit partial pericardial absence. We describe the histological changes and trace their developmental origin. We show that the two genes regulate distinct subprograms in PPM development, and define the cellular basis for PPC closure.

## Results

### *Tbx18*-deficient mice exhibit partial absence of the PPMs

**Figure 2:**
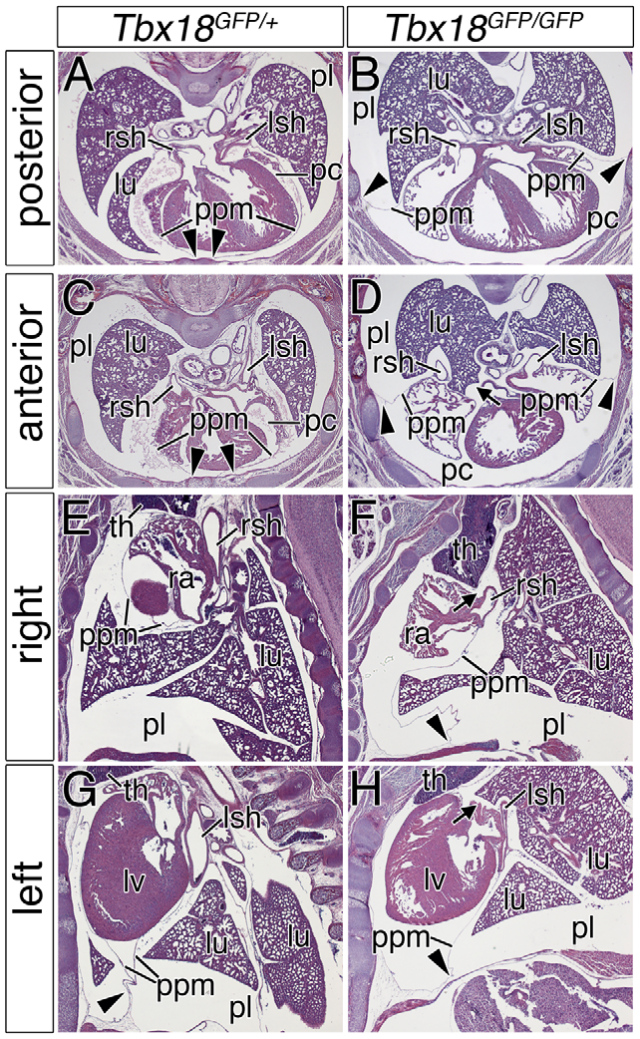
PPM defects in *Tbx18*-mutant embryos. (A–H) Histological stainings with haematoxylin and eosin were performed on transverse (A–D) and sagittal (E–H) sections of the venous pole region of the heart at E18.5. They reveal that PPMs are only partly released from the lateral body wall and that they are not attached to the mediastinum resulting in bilateral communication between the pleural and pericardial cavities in the mutant. Arrowheads mark the attachment point of the PPMs to the lateral body wall; arrows highlight the persisting PPC in *Tbx18*-deficient embryos. lu, lung; lsh, left sinus horn; lv, left ventricle; pc, pericardial cavity; pl, pleural cavity; ppm, pleuropericardial membrane; ra, right atrium; rsh, right sinus horn; th, thymus.

We recently demonstrated the importance of *Tbx18* for the development of the venous pole of the heart including the positioning and myocardialization of the sinus horns, the myocardial parts of the right and left superior cardinal veins within the pericardial cavity [6]. Our further analysis of *Tbx18*-mutant newborn mice revealed additional, as yet undescribed, pericardial defects that prompted us to investigate the role of *Tbx18* in the development of this tissue in more detail. We started our analysis by histological stainings of the thorax region at E18.5 (Figure 2), as *Tbx18*-null mice die shortly after birth [8]. In wildtype and control embryos (genotype: *Tbx18^GFP/+^*), the PPMs presented as thin epithelial sheets, which completely surrounded the heart. They were attached dorsally to the hilus of the lung and ventrally to the sternum in both posterior and anterior transverse sections through the embryo (Figure 2A and C, arrowheads). The right and left sinus horns were positioned medially, directly adjacent to the hilus of the lung (Figure 2A and C). In contrast, the PPMs of *Tbx18*-deficient embryos (genotype: *Tbx18^GFP/GFP^*) only partially encircled the heart, as they were attached to the lateral body wall and not to the sternum (Figure 2B and D, arrowheads). The anterior aspect of the PPMs was not tethered to the hilus of the lungs, allowing the lung to touch the atria (Figure 2D, arrow). Furthermore, the right and left sinus horns were located abnormally laterally and within the PPMs at this stage (Figure 2B and D). Sagittal sections confirmed the complete separation of the pleural and pericardial cavities in control embryos (Figure 2E and G). In *Tbx18*-deficient embryos pericardial and pleural cavities communicated in the dorsal hilus region (Figure 2F and H, arrows). Partial absence of the PPMs occurred bilaterally and was fully penetrant (n = 12). Moreover, the posterior aspect of the PPMs was attached to the diaphragm in an abnormal dorsal position (Figure 2F and H, arrowheads). Together, these data indicate that *Tbx18* is required for the lateral release and/or ventral and dorsal attachment of the PPMs.

### PPM development is severely disturbed in *Tbx18*-deficient embryos

**Figure 3:**
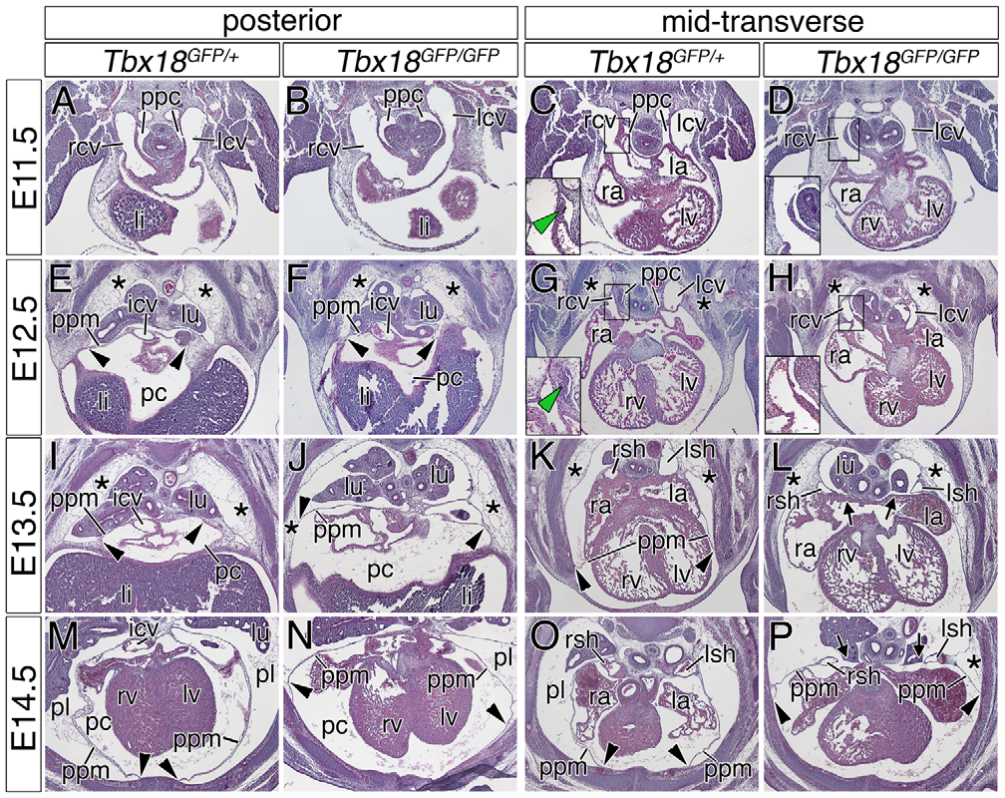
PPM development in control and *Tbx18*-deficient embryos. (A–P), Histological analysis by haematoxylin and eosin stainings of PPM development was performed on transverse sections of a posterior and a mid-transverse section plane of control and *Tbx18*-deficient hearts from E11.5 to E14.5. Magnifications (C, D, G, H) highlight the sinuatrial mesenchymal ridges that protrude into the PPCs in control embryos at E11.5 and E12.5, and their absence in *Tbx18*-deficient embryos, respectively. Genotypes and stages are as indicated. Asterisks highlight the subcoelomic lateral body wall mesenchyme; black arrowheads mark the attachment point of the PPMs to the lateral body wall; black arrows point to the remaining PPCs; and green arrowheads mark the mesenchymal ridge that is necessary for the closure of the PPCs. icv, inferior cardinal vein; la, left atrium; lcv, left cardinal vein; li, liver; lu, lung; lsh, left sinus horn; lv, left ventricle; pc, pericardial cavity; ppc, pericardioperitoneal canal; pl, pleural cavity; ppm, pleuropericardial membrane; ra, right atrium; rcv, right cardinal vein; rsh, right sinus horn; rv, right ventricle.

In order to investigate the development of PPMs, and to determine the onset of pericardial defects in *Tbx18*-deficient mice, we prepared serial histological stainings through the thoracic region of E9.5 to E14.5 control and *Tbx18*-mutant embryos. We present transverse sections from both a posterior and a middle level (Figure 3), and parasagittal sections (Figure S1) to illustrate the positions of the lung, the cardinal veins, and the PPMs relative to each other at the cardiac venous pole.

At E9.5 and E10.5, the right and left cardinal veins drained into the common atrium at a position posterior to the heart. The lung buds expanded into the PPCs and were in direct contact to the atrium in both control and *Tbx18*-mutant embryos (data not shown). At E11.5, the inflow tract (IFT) of the heart including the right and left cardinal vein was still positioned at a posterior position in the thoracic cavity, and the subcoelomic mesenchyme next to the developing cardinal veins was tightly organized in both genotypes (Figure 3A and C). Mesenchymal tissue ridges protruded bilaterally at the border of atrium and sinus horns into the PPCs of control embryos, but were absent in the mutant heart (Figure 3C and D, Figure S1A and F, marked by a green arrowhead). At E12.5, the IFT was no longer detectable on a posterior section plane through the thoracic cavity, but was positioned on mid-transverse levels in both genotypes. PPMs, which were distally attached to the ventrolateral body wall but not to the dorsal mesocardium, were visible at posterior levels of the heart, next to the IFT, at this developmental time point but not in more anterior sections (Figure 3E–H). The mesenchymal ridges at the sinuatrial/PPM border were expanded and largely compressed the lumen of the PPCs at a dorsomedial position in control embryos (Figure 3G, Figure S1B, green arrowhead). *Tbx18*-deficient embryos completely lacked these mesenchymal ridges, the PPCs appeared broader, and the sinus horns were positioned more laterally in mid-transverse sections (Figure 3H, Figure S1G).

Starting from E12.5, the lateral contact points of the PPMs with the body wall were displaced ventrally following the distal extension of the ribs in control embryos; mediodorsally, the PPMs became attached to the hilus of the lung between E12.5 and E13.5. Pleural and pericardial cavities were completely separated by PPMs in control mice at E13.5 (Figure 3K, Figure S1C). The subcoelomic mesenchyme in the pleural cavity and around the common cardinal veins became extremely loosely organized starting from E12.5, and was almost completely vanished in control embryos at E14.5 (marked by asterisks in Figure 3I–P). In *Tbx18*-mutant embryos, the loose subcoelomic mesenchyme was still present next to the sinus horns at E13.5 and E14.5 (Figure 3L and P, asterisks). The sinus horns were embedded in the PPMs and positioned much more laterally on both sides of *Tbx18*-deficient hearts starting from E13.5 until E18.5 (Figures 3L and P). By E14.5, the PPMs were stretched into thin epithelial sheets and connected to the sternum in wildtype embryos (Figure 3M and O, arrowheads). In *Tbx18*-mutant embryos the PPMs were not connected to the sternum but were fixed to a lateral position (Figure 3N and P, arrowheads). Furthermore, the PPCs were not closed and the dorsal attachment of the PPMs to the hilus of the lung was absent in transverse sections on mid and anterior levels through the thoracic region of *Tbx18*-deficient embryos, leading to a partial bilateral absence of the PPMs in these embryos (Figure 3L and P, arrows, Figure S1H and I). Therefore, failure of formation of sinuatrial mesenchymal ridges may underlie the observed defects in PPM and sinus horn development in *Tbx18*-deficient embryos.

### *Tbx18* is expressed in the developing sinus horns and in adjacent dorsal mesenchymal ridges

**Figure 4:**
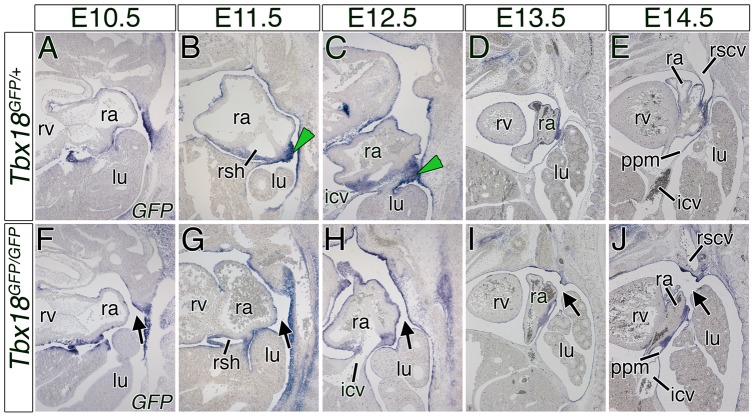
The PPCs in *Tbx18*-deficient embryos remain open due to the loss of *GFP*-positive sinuatrial mesenchymal ridges. (A–J) *In situ* hybridization analysis of *GFP* expression on sagittal sections of control and mutant hearts from E10.5 to E14.5. In *Tbx18*-deficient embryos the mesenchymal ridges are not established (F–J). Stages are as indicated on top and the genotypes on the left side. Black arrows point to the open right PPC. Green arrowheads mark the *GFP*-positive mesenchymal ridges in control embryos at E11.5 and E12.5. icv, inferior cardinal vein; lu, lung; ppm, pleuropericardial membrane; ra, right atrium; rscv; right superior caval vein; rsh, right sinus horn; rv, right ventricle.

To further analyze the role of *Tbx18* in PPM development, we analyzed expression of this gene at the cardiac venous pole from E10.5 through E14.5 by *in situ* hybridization. To be able to compare the wildtype with the *Tbx18* loss-of-function situation, we analyzed expression of the integrated reporter gene *green fluorescent protein* (*GFP*) in *Tbx18^GFP/+^* (control) and *Tbx18^GFP/GFP^* (mutant) embryos (Figure 4). In agreement with previous reports, we identified expression of *Tbx18*/*GFP* in the epicardium, in the parietal layer of the serous membrane lining the wall of the thoracic cavity, in the PPMs, and in the sinus horn mesenchyme and myocardium in control embryos throughout these stages (Figure 4A–E) [6], [9]. We newly discovered strong expression of *Tbx18*/*GFP* in the mesenchymal ridges at the border between the sinus horns, the atria, and the PPCs from E11.5 to E12.5 (Figure 4B and C). Obviously, lack of this tissue in the mutant situation did not permit to check for maintenance of expression of *Tbx18*/*GFP*, respectively. However, all other expression domains of *Tbx18* were unaffected as reflected by the expression of *GFP* in *Tbx18*-deficient embryos at all analyzed stages (Figure 4F–J). This strongly indicates that *Tbx18* is cell-autonomously required for the formation of the mesenchymal ridges at the sinus horns.

### Partial absence of PPMs in *Wt1*-deficient mice

**Figure 5:**
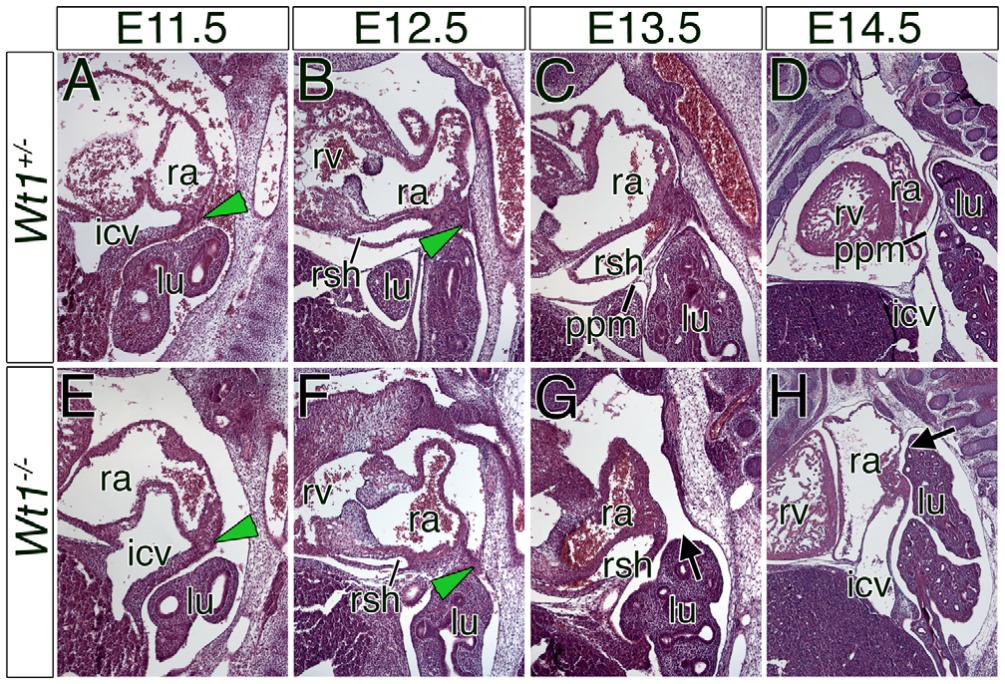
PPC closure is disturbed in *Wt1*-deficient embryos. (A–H) Histological analysis by haematoxylin and eosin stainings on sagittal sections through the PPCs of control (genotype: *Wt1^+/−^*) and *Wt1*-mutant embryos from E11.5 to E14.5. Black arrows highlight the pericardial defect in *Wt1*-deficient embryos at E13.5 and E14.5. Green arrowheads mark the mesenchymal ridges in control and Wt1-deficient embryos at E11.5 and E12.5. Stages and genotypes are as indicated. icv, inferior cardinal vein; lu, lung; ppm, pleuropericardial membrane; ra, right atrium; rsh, right sinus horn; rv; right ventricle.

We recently demonstrated the importance of the transcription factor Wt1 and retinoic acid signaling in the subcoelomic mesenchyme for the ventral displacement of the lateral PPM attachment points in the thoracic cavity, thus, for the expansion of the pleural cavity [5]. Since *Wt1*-deficient mice also displayed pleuropericardial communication and a lateral displacement of the cardinal veins, we wondered whether a defect in PPC closure might contribute to this phenotype. Histological analysis of sagittal sections through the thoracic region of *Wt1*-deficient embryos revealed sinuatrial mesenchymal ridges at E11.5, and dramatically narrowed PPCs at E12.5 as in the control embryos (genotype: *Wt1^+/−^*) (Figure 5A and B, E and F, green arrowheads). The PPCs were completely obliterated in the E13.5 control embryo, whereas they were grossly widened at this stage in the *Wt1*-mutant embryo (Figure 5C and G). At E13.5 and E14.5, we observed pleuropericardial communication in the medial region (Figure 5G and H, black arrows), and lateralized sinus horns embedded in truncated PPMs in 6 out of 7 specimens. In four cases the defect was restricted to the right side, in two cases to the left side. In the control, the pleural and pericardial cavities were completely separated at this stage (Figure 5C and D).

Histological analysis on transverse sections through the thoracic region confirmed that the sinuatrial mesenchymal ridges were formed and contacted the lung hilus at E12.5 but did not fuse with it in *Wt1*-deficient embryos. These analyses also revealed that the cardinal veins were fixed to the lateral body wall by a mesh of loose mesenchymal cells, and pleural and pericardial cavities communicated at E13.5 and E14.5 (Figure S2).

Our analysis suggests that partial PPM absence in *Wt1*-deficient embryos is caused by a failure of the sinuatrial mesenchymal ridges to fuse with the mesothelial ling of the lung hilus. The lacking ventral displacement of the attachment of the PPMs along the lateral body wall may be interconnected.

### The sinuatrial mesenchymal ridges are highly proliferative

**Figure 6:**
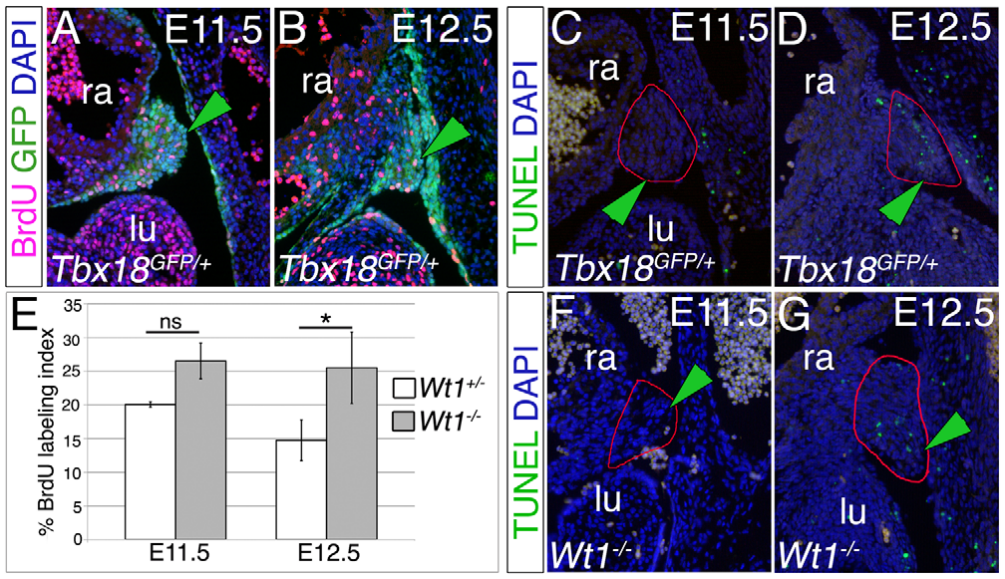
Sinuatrial mesenchymal ridges are highly proliferative. (A–D) Immunohistochemical analysis for GFP protein (green) and BrdU (red) (A, B) and the TUNEL assay (C, D) on sagittal sections through the heart of *Tbx18^GFP/+^* embryos at E11.5 and E12.5 show that the mesenchymal ridges (green arrowheads) are highly proliferative and that apoptosis is absent. (E) Quantitative analysis of proliferation by the BrdU assay reveals non-significant changes of proliferation at E11.5 and significantly increased proliferation in *Wt1*-deficient sinuatrial ridges at E12.5. (F, G) The TUNEL assay on sagittal sections through the heart of *Wt1^−/−^* at E11.5 and E12.5 shows that apoptosis is absent from mesenchymal ridges (green arrowheads) at these stages. lu, lung; ra, right atrium.

To achieve further insight into the cellular mechanism of PPC closure, we analyzed proliferation and apoptosis in the sinuatrial mesenchymal ridges in “wildtype” embryos at E11.5 and E12.5 (Figure 6). To unambiguously identify this structure we performed co-immunofluorescence analysis against GFP protein in *Tbx18^GFP/+^* embryos. The BrdU incorporation assay depicted a high number of proliferating cells in the GFP-positive mesenchymal ridges in these control embryos at E11.5 and E12.5 (marked by a green arrowhead in Figure 6A and B). This suggests that the increase in cell number in the ridges contributes to constriction of the PPCs. Analysis of cell death by the TUNEL assay did not detect apoptotic cells inside the mesenchymal ridges at E11.5, but a small number of these cells were observed in this structure at E12.5 (Figure 6C and D). As mentioned before, these GFP-positive mesenchymal ridges were absent in *Tbx18*-mutant embryos (Figure 4G–J, arrows) precluding the analysis of proliferation and apoptosis. In *Wt1*-deficient embryos we detected slightly (E11.5) and significantly increased proliferation at E12.5, but unchanged apoptosis in the sinuatrial ridges at these stages (Figure 6E–G, Figure S3). The functional relevance of increased proliferation is unclear.

### No cross-regulation of *Tbx18* and *Wt1* in the sinuatrial mesenchymal ridges

**Figure 7:**
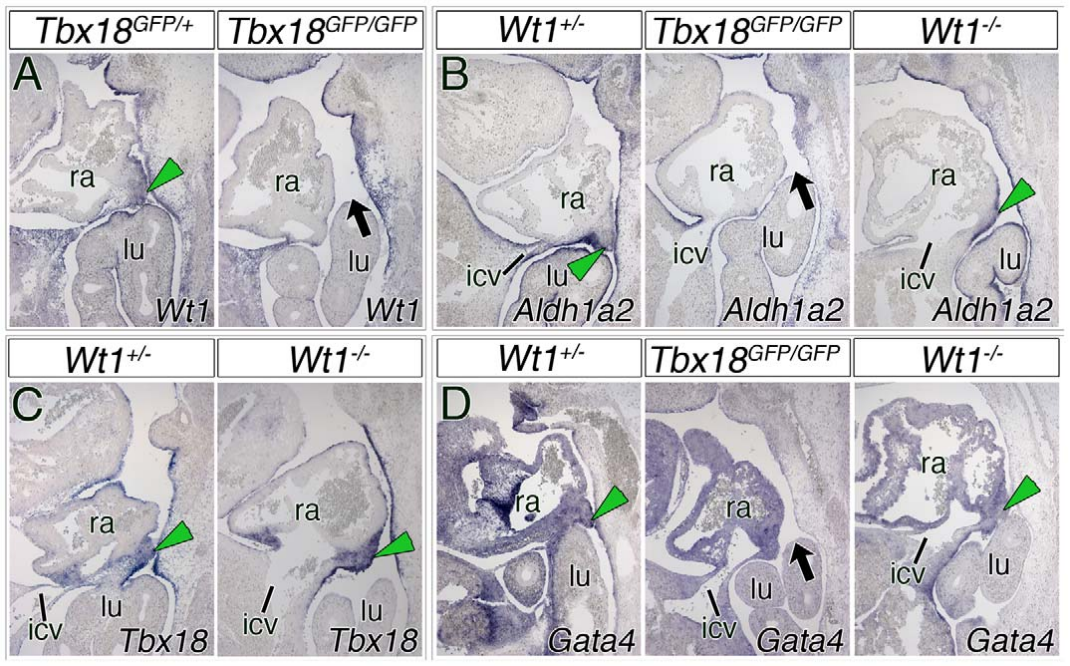
Gene expression in and around the sinuatrial ridges in *Wt1*- and *Tbx18*-mutant hearts. (A–D) *In situ* hybridization analysis of *Wt1* (A), *Aldh1a2* (B), *Tbx18* (C) and *Gata4* (D) on sagittal sections trough the venous pole region of control, *Wt1*- and *Tbx18*-deficient hearts at E11.5 and E12.5. Genotypes and stages are as indicated. Green arrowheads point to the mesenchymal ridges that are important for the closure of the PPCs in control embryos; black arrows mark the remaining PPCs. icv, inferior cardinal vein; lu, lung; ra, right atrium.

Given the findings that loss of both *Tbx18* and *Wt1* affects the development of the sinuatrial mesenchymal ridges, and leads to PPC persistence, we wondered whether the two genes are part of a common pathway or act independently. Therefore, we examined the expression of *Wt1* as well as of *Aldh1a2* (also known as *Raldh2*) that are found in the subcoelomic mesenchyme, the mesothelial lining covering the pleural and pericardial cavities, and the epicardium [5] but not in the sinuatrial ridges themselves. Of course, absence of these ridges in *Tbx18*-deficient embryos does not allow the analysis of gene expression within these domains. However, we found normal expression of *Wt1* and *Aldh1a2* in adjacent mesothelial linings and the subcoelomic mesenchyme of *Tbx18*-deficient embryos (Figure 7A and B), suggesting that *Tbx18* does not regulate these genes. The morphology of the sinuatrial region is disturbed in *Wt1*-deficient embryos [5]. Nonetheless, we detected *Tbx18*-positive sinuatrial mesenchymal ridges in *Wt1*-deficient embryos at E11.5, E12.5 and E13.0 confirming that Wt1 does neither directly nor indirectly regulate *Tbx18* expression in this domain (Figure 7C, Figure S4). We finally analyzed expression of *Gata4* that was previously implicated in pericardial development [10]. Expression of *Gata4* within the ridge was unchanged in *Wt1*-deficient embryos as it was in the atrial and sinus horn myocardium in *Tbx18*-mutant embryos (Figure 7D).

We conclude that *Wt1* and *Tbx18* expression in the sinuatrial region, the mesothelial lining and the subcoelomic mesenchyme are independent of each other, and that they regulate distinct subprograms in the formation of sinuatrial ridges to obliterate the PPCs.

## Discussion

Absence of the PPMs is a rare congenital human defect with unclear molecular and cellular origin. Here, we presented two mouse mutants with bilateral (*Tbx18*) or unilateral (*Wt1*) partial PPM absence in the dorsomedial region. We have traced these defects to a failure to establish sinuatrial mesenchymal ridges (*Tbx18^−/−^* mice) and to fuse them with the lining of the dorsal body wall (*Wt1*-deficient embryos). We discuss the interrelation between lateral PPM release, sinus horn morphogenesis, and PPC closure.

### *Tbx18*-positive sinuatrial mesenchymal ridges are required for attaching the PPMs to the mediastinum

PPCs are bilateral communication areas that arise in the early embryo by partial partitioning of the coelomic cavity. At their anterior end, they initially allow the growth and descent of the emerging lung bud. However, complete separation of the pleural, pericardial and peritoneal cavities requires that pleuropericardial and pleuroperitoneal membranes are formed that eventually obliterate these canals. In the human embryo, it was suggested that initially two folds grow out of the body wall, the ventral one forming the pleuropericardial, and the dorsal one forming the pleuroperitoneal membrane. These folds elongate and eventually reach the medial wall of the pleuroperitoneal canals on each side of the lung bud. Attachment of the PPMs and the embedded cardinal veins with the lung hilus leads to complete separation of the pleural and pericardial cavities [1]. The mechanisms that drive this process have remained obscure. It was suggested that the active growth of neighboring structures diminishes the size of the opening, that active growth mechanisms within the PPMs occur, and that the growing and turning cardinal veins passively close the PPCs [11].

**Figure 8:**
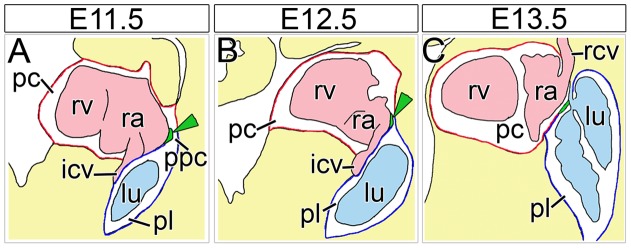
Schematic diagram of PPC closure in mouse development. (A–C) Scheme of PPC closure by sinuatrial mesenchymal ridges based on sagittal sections through the cardiac venous pole of wildtype embryos from E11.5 to E13.5. Sinuatrial mesenchymal ridges protrude into the PPCs at E11.5 and E12.5 and fuse with the overlying wall of the lung hilus at E13.5 to separate pleural and pericardial cavities. The lumen of the caval veins and the heart is marked in pale pink, the lung and trachea in pale blue, the mesenchymal sinuatrial ridges in green (additionally marked by green arrowheads), the (parietal) pericardium in red, the (parietal) pleura in dark blue and the double-walled PPMs in red and blue. icv; inferior caval vein; lu, lung; pc, pericardial cavity; pl, pleural cavity; ppc, pericardioperitoneal canal; ppm, pleuropericardial membranes; ra, right atrium; rcv, right superior caval vein; rv, right ventricle.

Our serial histological analysis of mouse embryos detected ridges as extension of the sinus horn mesenchyme and the associated PPMs around E11.5. During the next two days, these mesenchymal ridges expanded towards the lung hilus and narrowed the lumen of the initially broader pleuropericardial communication to a thin canal. Around E13.5, the mesothelial linings of the ridges and the lung bud fused, completely sealing the pleural from the pericardial cavity (schematized in Figure 8). The existence of such mesenchymal ridges that are crucial for PPC closure was noted in human embryos as well. By careful analysis of serially sectioned human embryos Salzer reported on such a “Mesenchymfalte” at the border of the Ductus Cuvieri and the PPM, and suggested that closure of the PPC occurs by an active mechanism rather than by mere passive growth of the neighboring structures in the region of the lung hilus. He argued that the final step of PPC closure represents a real fusion process between neighboring membranes that were juxtaposed by the previous expansion of the ridges and the overlying lung hilus [4]. This study may have remained unnoticed in the field due to its German language.

In *Tbx18*-deficient embryos these mesenchymal ridges were not established, and as a consequence PPC closure failed. This defines these mesenchymal ridges as distinct molecular entities, and confirms their functional relevance for the formation of complete PPMs. At this point, the precise cellular role of Tbx18 in the formation of these ridges remains open. Possibly, Tbx18 is required for establishment of a precursor pool similar to its role in the adjacent sinus horns [6]. Alternatively or additionally, Tbx18 may confer a high enough proliferation rate for quick expansion of the progenitor pool.

Failure of PPC closure in *Tbx18-*deficient embryos at E13.5 left the PPMs with only one attachment point at the lateral body wall. As a consequence of the tension exerted by the growing thoracic cavity, the pleuropericardial communication may widen again, by which, in turn, the cardinal veins were pulled laterally. Therefore, the incomplete detachment from the lateral body wall in *Tbx18*-mutant embryos might to some extent be a secondary effect of the lack of medial attachment of the cardinal veins to the lung hilus, and not a primary effect caused by cellular changes in the dorsolateral mesenchyme, since marker genes for the subcoelomic mesenchyme (*Wt1* and *Aldh1a2*) were not affected by the loss of *Tbx18*.

Patients with congenital defects of the PPMs most frequently present a complete left-sided absence of this tissue whereas right-sided or bilateral deficiencies are more rare. In *Tbx18*-deficient embryos we only detected bilateral and partial PPM absence in the dorsomedial region. We cannot exclude that postembryonic growth processes or degeneration would further increase the severity of the described defect. However, the preferential left-sided PPM absence in human patients may not relate to loss or reduction of the mesenchymal ridges, thus diminished or lost expression of *Tbx18*, since this is likely to occur bilaterally. Premature atrophy of the left duct of Cuvier as suggested by Perna (1909) [12] or excessive growth of the left-side of the heart may account for these cases [11].

### Failure to close the PPCs in *Wt1-*deficient embryos may be secondary to defects in PPM release from the lateral body wall

Our serial histological analysis of mouse embryos from E9.5 to E14.5 did not detect “folds” growing out of the lateral body wall and pointing into the coelomic cavity, as sometimes described for human embryos [1]. We noted PPMs first at the posterior aspect of the heart at E12.5. They always presented as sheets harboring the inferior cardinal vein at the posterior end, and slightly later the superior cardinal veins at more anterior levels. After the PPMs were connected to the hilus of the lung, highly dynamic and complex morphogenetic changes occurred that expanded the PPMs and progressively shifted their lateral attachment point from dorsal to ventral. This resulted in a massive expansion of the pleural at the expense of the pericardial cavity (schematized in Figure 1). Parallel to this process the lungs massively grew and the thoracic cavity enlarged dramatically. Although early studies suggested that lung growth is a driving force in this process, a normally sized pleural cavity in absence of a lung excluded this hypothesis [13]. Salzer (1960) pointed to the possibility that the expansion of the thoracic cavity and the growth of the ribs may be more relevant, and that in fact the lateral attachment points of the PPMs are fixed and are passively pulled ventrally, thereby inducing growth of the PPMs [14]. He contradicted earlier descriptions of Uskow (1883) [15] who suggested that the PPMs are progressively released from the mesothelial lining, the parietal pleura, of the lateral body wall in a dorsal to ventral fashion. Frick (and later Salzer) noted the transient existence of the loose mesenchymal mesh that they interpreted as a “placeholder” for the pleural cavity [14], [16].

Our previous and current analysis of *Wt1*-deficient embryos [5] argue against a passive elongation and ventral displacement of the PPMs but suggests that PPMs are progressively released by a tightly controlled mechanism and that the subcoelomic mesenchyme plays a crucial role therein. First, the subcoelomic mesenchyme next to the cardinal veins becomes loosely organized and disappears completely between E12.5 and E14.5, a process that is tightly coupled with the dorsoventral release of the PPMs, and the dramatic increase in the size of the pleural cavities and the lung. Second, we recently showed that *Wt1* and its target gene *Aldh1a2* are required within the subcoelomic body wall mesenchyme for the detachment of the PPMs. Retinoic acid induced apoptosis is absent in the *Wt1*-deficient embryos and therefore the subcoelomic mesenchyme persists. The PPMs are not released form the lateral body wall, the caval veins become positioned abnormally laterally [5].

Our present study revealed that closure of the PPCs is affected in *Wt1*-deficient embryos as well. In contrast to *Tbx18*-mutant mice, the sinuatrial mesenchymal ridges are established and the pleuropericardial communications are narrowed to very thin canals at E13.0. However, the fusion of two mesothelial surfaces of the ridge and the overlying tracheal wall failed. Although we cannot exclude the possibility that *Wt1* is required within the mesothelial lining to mediate the final fusion step, we assume that the lack of PPC closure does not reflect a primary requirement of *Wt1* in these tissues. Circumstantial evidence is provided by the randomization of the defect that can occur left or right, but was never observed bilaterally. We suggest that the persistence of the lateral body wall mesenchyme and the failure of PPM release concomitant with the massive expansion of the thoracic cavity result in tensional forces that physically prevent PPC closure, lead to lateralization of the cardinal veins and partial PPM absence. Non-attachment of the PPMs to the lung hilus may further negatively impact on the non-release from the lateral body wall. Independent from a primary or secondary nature, it may be interesting to analyze in the future whether the fusion process between the membranes of the sinuatrial ridges and the overlying tissue mechanistically resembles other fusion processes in the embryo as e.g. seen in the palate and the paired dorsal aorta.

## Materials and Methods

### Ethics statement

All animal work conducted for this study was approved by H. Hedrich, state head of the animal facility at Medizinische Hochschule Hannover and performed according to German legislation.

### Mice and genotyping

Transgenic mice harboring a knock-in of the GFP reporter in the *Tbx18* locus allele (*Tbx18^tm2Akis^*, synonym: *Tbx18^GFP^*) [7] and mice carrying a null allele of *Wt1* (*Wt1^tm1Jae^*) [17] were described before. Heterozygous embryos (*Tbx18^GFP/+^* or *Wt1^+/−^*) were used as control embryos. All mice were maintained on an outbred (NMRI) background. Mice were kept with regulated temperature (18–22°C) and humidity (∼50%) with a 12 h light/dark cycle. For the generation of mutant embryos, heterozygous mice were intercrossed. Vaginal plugs were checked in the morning after mating, for timed pregnancies noon was taken as E0.5. Female mice were sacrificed by cervical dislocation. Embryos were harvested in PBS, decapitated, fixed in 4% paraformaldehyde overnight and stored in 100% methanol at –20°C before further use. Genomic DNA prepared from yolk sacs or tail biopsies was used for genotyping by PCR.

### Histological analysis

Embryos were fixed as mentioned above, paraffin embedded, and sectioned to 10 µm for histological stainings. Sections were stained with haematoxylin and eosin following standard procedures. At least two embryos of each genotype were used for each analysis.

### *In situ* hybridization analysis

Nonradioactive *in situ* hybridization analysis with digoxigenin-labeled antisense riboprobes was performed as described [18]. At least two embryos of each genotype were used for each analysis. Details of used probes upon request.

### Immunohistochemistry, proliferation and apoptosis assays

Cell proliferation in embryonic day (E) 11.5 and E12.5 in *Wt1*-deficient mice was investigated by detection of incorporated 5-bromo-2-deoxyuridine (BrdU) on 5-µm sections of paraffin-embedded specimens similar to previously published protocols [8]. *Wt1-*heterozygous littermates were used as controls.

Immunohistochemical co-staining of BrdU and GFP was performed on 5-µm sections of *Tbx18^GFP^* heterozygous and mutant mice at E11.5 and E12.5. The primary anti-GFP antibody (rabbit polyclonal antibody against GFP, 1∶300, sc-8334, Santa Cruz) was amplified using the Tyramide Signal Amplification (TSA) system from Perkin-Elmer (NEL702001KT, Perkin Elmer LAS) and biotinylated goat–anti-rabbit (1∶250, 111-065-003, Jackson ImmunoResearch). BrdU was detected using a mouse monoclonal antibody against BrdU (1∶200, 1170376, Roche) as primary and Alexa488 donkey-anti-mouse (1∶250, Invitrogen) as secondary antibodies following the published protocols. Nuclei were stained with 4′,6-Diamidino-2-phenylindol (DAPI) (Roth). At least four sections of three embryos of each genotype were used for quantification at E11.5 and E12.5.

The rate of apoptotic cells on 10-µm paraffin sections was analyzed by performing the TUNEL-assay following the protocol provided by the manufacturer (Serologicals Corp.) of the ApopTag kit used. Four sections each of three control and *Wt1-* or *Tbx18*-deficient embryos were analyzed at E11.5 and E12.5.

### Image analysis

Sections were photographed using a Leica DM5000 microscope with Leica DFC300FX digital camera and afterwards processed in Adobe Photoshop CS3.

## Supporting Information

Figure S1 **Insufficient closure of the PPCs in** ***Tbx18-*** **mutant embryos.** (A–J) Histological analysis by haematoxylin and eosin staining was performed on sagittal sections of E11.5 to E14.5 control (upper row) and *Tbx18*-deficient (lower row) hearts as indicated. Arrows highlight the remaining PPCs in *Tbx18*-deficient embryos. Green arrowheads mark the sinuatrial ridges, black arrows point to the persisting PPCs in *Tbx18*-deficient embryos. lu, lung; oft, outflow tract; pc, pericardial cavity; pl, pleural cavity; ppm, pleuropericardial membrane; ra, right atrium; rsh, right sinus horn; rv, right ventricle; sh, sinus horn.
(TIF)

Figure S2 **Pericardial defects in** ***Wt1*** **-deficient hearts.** (A–H) Histological analysis by haematoxylin and eosin stainings on transverse sections through the PPCs of control (genotype: *Wt1^+/−^*) and *Wt1*-deficient embryos from E11.5 to E14.5. (I–L) *In situ* hybridization analysis of *Tbx18* expression on transverse sections trough the venous pole region of control and *Wt1*-deficient hearts at E11.5 (I, J) and E12.5 (K, L). Stages and genotypes are as indicated. Green arrowheads point to the sinuatrial ridges in both control and *Wt1*-deficient embryos. Black arrows highlight the PPM defects in *Wt1*-deficient embryos at E13.5 and E14.5. icv; inferior caval vein; la, left atrium; lsh, left sinus horn; lu, lung; ppm, pleuropericardial membrane; ra, right atrium; rv, right ventricle; rsh, right sinus horn.
(TIF)

Figure S3 **Proliferation analysis of the sinuatrial mesenchymal ridges in** ***Wt1-*** **deficient mice.** (A–D) Analysis of proliferation by BrdU immunohistochemistry in the red encircled domain of the sinuatrial region performed on sagittal sections through the PPCs at E11.5 and E12.5 identifies the sinuatrial ridges as a highly proliferative tissue. BrdU positive cells are labeled in green. Stages and genotypes are as indicated. Green arrowheads point to the mesenchymal ridges in control and *Wt1*-deficient embryos. lu, lung; ra, right atrium.
(TIF)

Figure S4 ***Tbx18*** **expression in and around the sinuatrial ridges in** ***Wt1*** **-mutant hearts.** (A–D) *In situ* hybridization analysis of *Wt1* expression on sagittal sections trough the venous pole region of control and *Wt1*-deficient hearts at E11.5 and E13.0. Genotypes and stages are as indicated. Green arrowheads point to the mesenchymal ridges that are also established in *Wt1*-deficient hearts. icv, inferior cardinal vein; lu, lung; ra, right atrium.
(TIF)
