# Supplementary figures and images for: Partial Absence of Pleuropericardial Membranes in Tbx18- and Wt1-Deficient Mice
Source: PLoS One. 2012 Sep 11;7(9):e45100. doi: 10.1371/journal.pone.0045100 (PMC3439432; doi:10.1371/journal.pone.0045100)

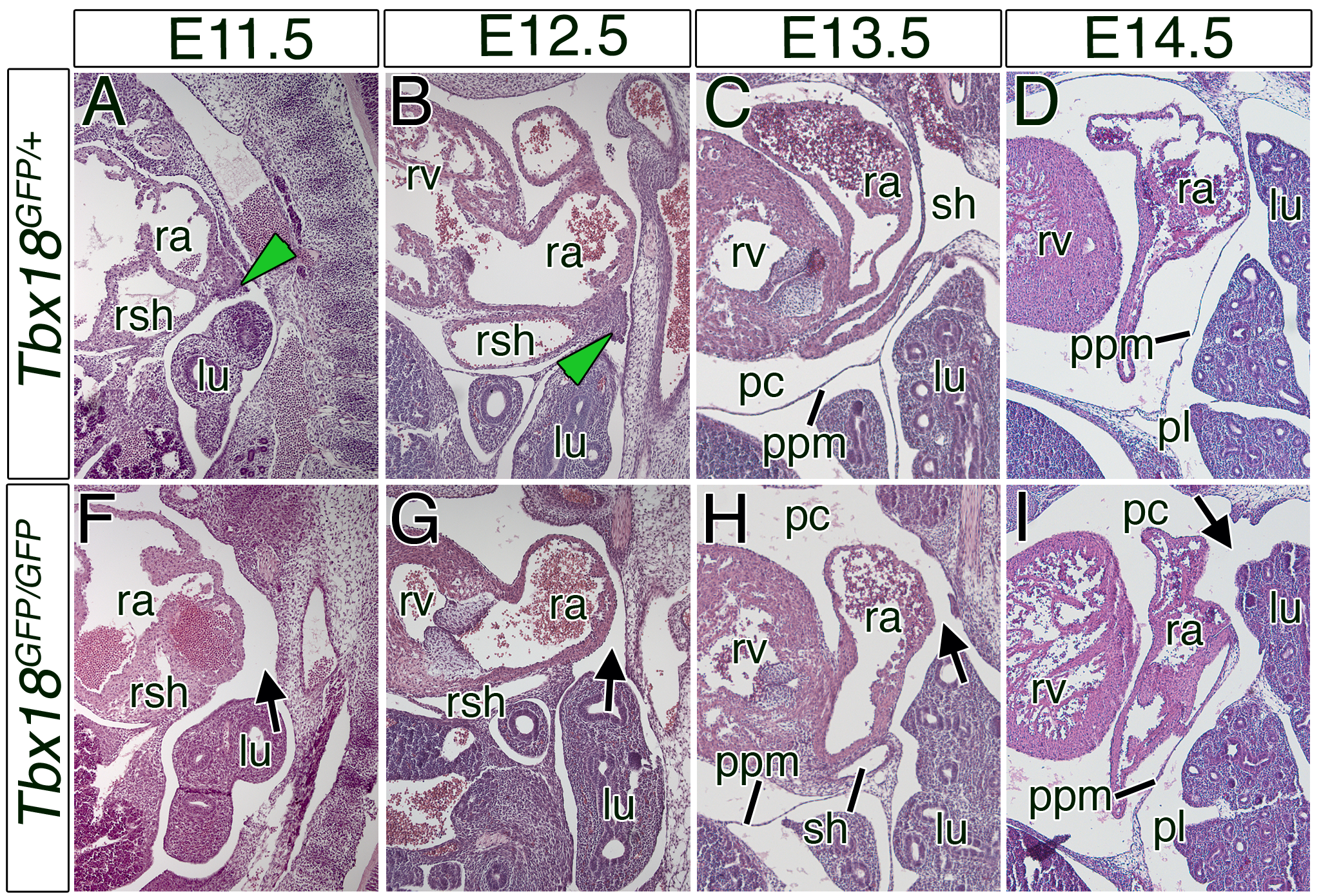

Supplement: Figure S1 — Insufficient closure of the PPCs in Tbx18- mutant embryos. (A–J) Histological analysis by haematoxylin and eosin staining was performed on sagittal sections of E11.5 to E14.5 control (upper row) and Tbx18-deficient (lower row) hearts as indicated. Arrows highlight the remaining PPCs in Tbx18-deficient embryos. Green arrowheads mark the sinuatrial ridges, black arrows point to the persisting PPCs in Tbx18-deficient embryos. lu, lung; oft, outflow tract; pc, pericardial cavity; pl, pleural cavity; ppm, pleuropericardial membrane; ra, right atrium; rsh, right sinus horn; rv, right ventricle; sh, sinus horn. (TIF) [file pone.0045100.s001.tif]

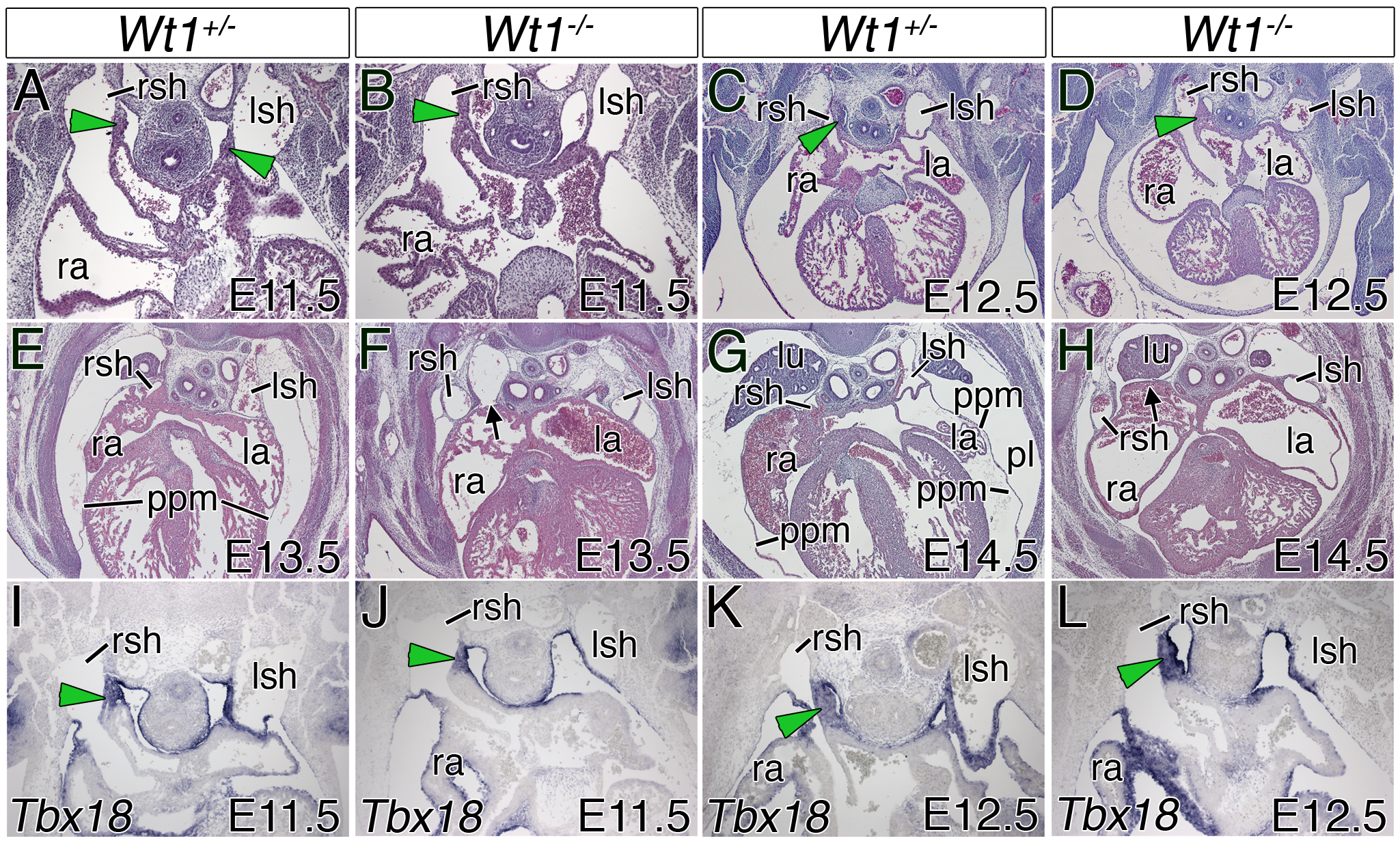

Supplement: Figure S2 — Pericardial defects in Wt1 -deficient hearts. (A–H) Histological analysis by haematoxylin and eosin stainings on transverse sections through the PPCs of control (genotype: Wt1+/−) and Wt1-deficient embryos from E11.5 to E14.5. (I–L) In situ hybridization analysis of Tbx18 expression on transverse sections trough the venous pole region of control and Wt1-deficient hearts at E11.5 (I, J) and E12.5 (K, L). Stages and genotypes are as indicated. Green arrowheads point to the sinuatrial ridges in both control and Wt1-deficient embryos. Black arrows highlight the PPM defects in Wt1-deficient embryos at E13.5 and E14.5. icv; inferior caval vein; la, left atrium; lsh, left sinus horn; lu, lung; ppm, pleuropericardial membrane; ra, right atrium; rv, right ventricle; rsh, right sinus horn. (TIF) [file pone.0045100.s002.tif]

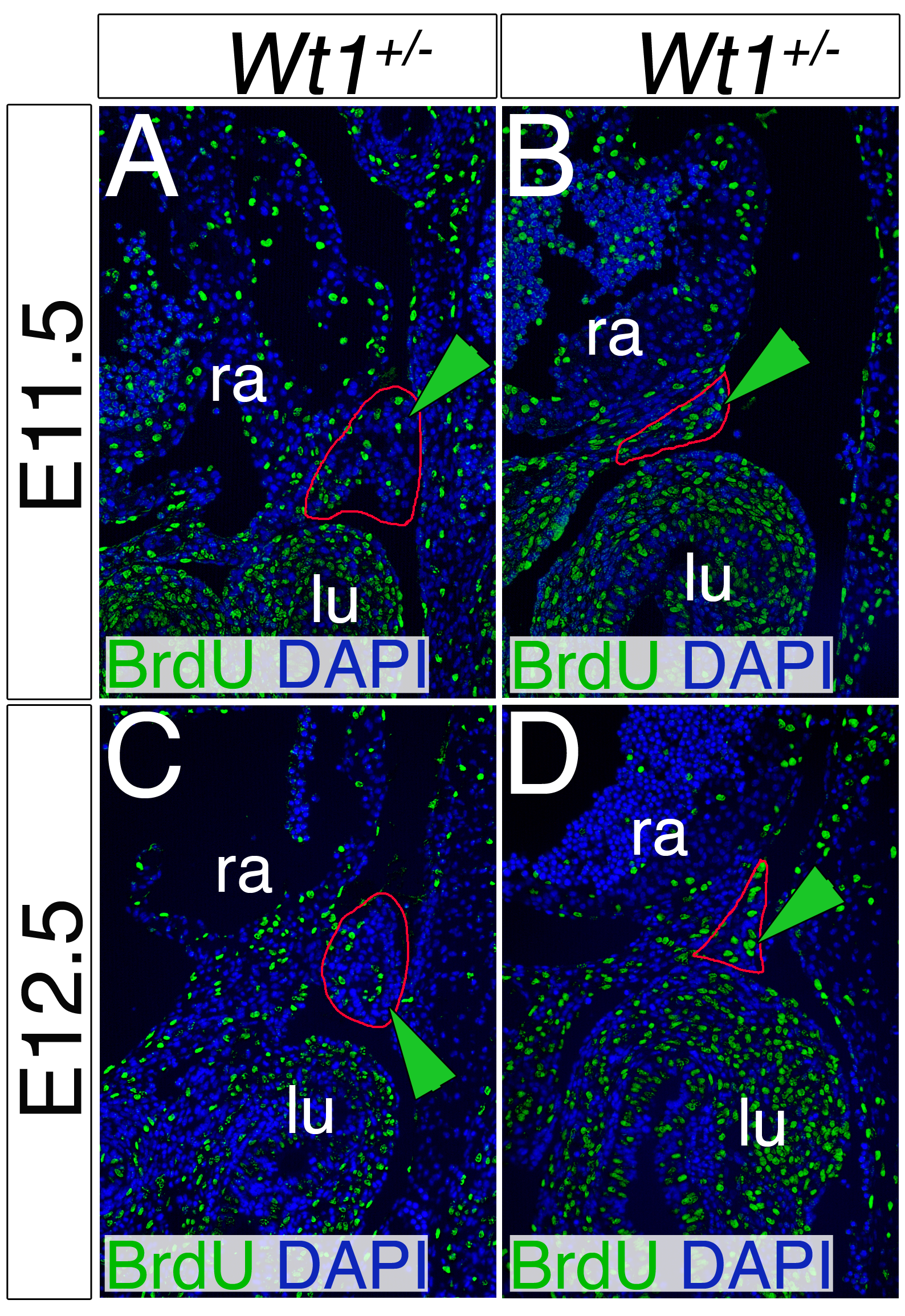

Supplement: Figure S3 — Proliferation analysis of the sinuatrial mesenchymal ridges in Wt1- deficient mice. (A–D) Analysis of proliferation by BrdU immunohistochemistry in the red encircled domain of the sinuatrial region performed on sagittal sections through the PPCs at E11.5 and E12.5 identifies the sinuatrial ridges as a highly proliferative tissue. BrdU positive cells are labeled in green. Stages and genotypes are as indicated. Green arrowheads point to the mesenchymal ridges in control and Wt1-deficient embryos. lu, lung; ra, right atrium. (TIF) [file pone.0045100.s003.tif]

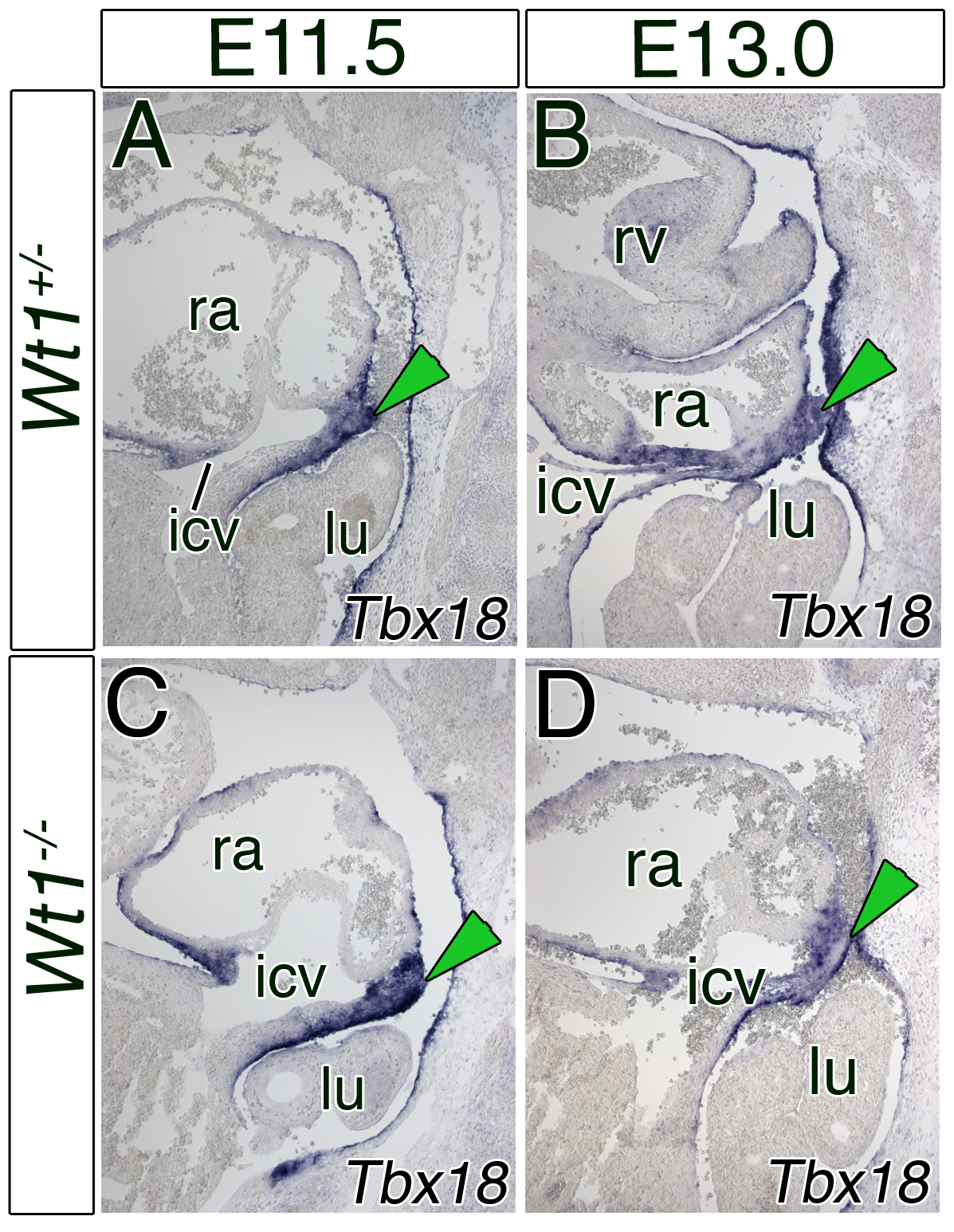

Supplement: Figure S4 — Tbx18 expression in and around the sinuatrial ridges in Wt1 -mutant hearts. (A–D) In situ hybridization analysis of Wt1 expression on sagittal sections trough the venous pole region of control and Wt1-deficient hearts at E11.5 and E13.0. Genotypes and stages are as indicated. Green arrowheads point to the mesenchymal ridges that are also established in Wt1-deficient hearts. icv, inferior cardinal vein; lu, lung; ra, right atrium. (TIF) [file pone.0045100.s004.tif]
